# Mechanisms Suggesting a Relationship between Vitamin D and Erectile Dysfunction: An Overview

**DOI:** 10.3390/biom13060930

**Published:** 2023-06-01

**Authors:** Andrea Crafa, Rossella Cannarella, Federica Barbagallo, Claudia Leanza, Roberto Palazzolo, Hunter Ausley Flores, Sandro La Vignera, Rosita A. Condorelli, Aldo E. Calogero

**Affiliations:** 1 Department of Clinical and Experimental Medicine, University of Catania, 95121 Catania, Italy; crafa.andrea@outlook.it (A.C.); federica.barbagallo11@gmail.com (F.B.); claudia.leanza.95@gmail.com (C.L.); robertopalazzolomed@gmail.com (R.P.); sandrolavignera@unict.it (S.L.V.); rosita.condorelli@unict.it (R.A.C.); aldo.calogero@unict.it (A.E.C.); 2 Glickman Urological & Kidney Institute, Cleveland Clinic Foundation, Cleveland, OH 44195, USA; 3 Scott Department of Urology, Baylor College of Medicine in Houston, Houston, TX 77030, USA; hunter.flores@cuanschutz.edu

**Keywords:** vitamin D, vitamin D deficiency, erectile dysfunction, endothelial dysfunction, hypertension, hypogonadism, dyslipidemia, chronic kidney disease

## Abstract

Vitamin D deficiency (VDD) and erectile dysfunction (ED) heavily burden the male population. The higher prevalence of both conditions in the elderly suggests a possible relationship between the two conditions. In addition, in vitro, animal, and human studies have revealed several mechanisms that may relate VDD to ED. The main mechanism by which vitamin D might exert its action on sexual function appears to be through the regulation of endothelial function. Indeed, VDD correlates with several markers of endothelial function. The action of vitamin D on the endothelium would be exercised both indirectly through its intervention in inflammatory processes and through the production of oxygen free radicals, and directly through the regulation of vascular stiffness, the production of nitric oxide, and the regulation of vessel permeability. Furthermore, the ubiquitous distribution of the vitamin D receptor in the human body means that this hormone can also exert a beneficial effect on erectile function by interfering with those comorbidities significantly associated with ED, such as hypertension, diabetes mellitus, hypercholesterolemia, chronic kidney disease, and hypogonadism. In this review, we thoroughly and carefully presented the evidence and mechanisms that would appear to relate vitamin D levels to erectile function. Furthermore, we have summarized the meta-analytic evidence for and against this association to provide a true representation of this topic. Data published to date suggest that low levels of vitamin D could contribute to worsening erectile function through several mechanisms. Therefore, vitamin D levels should be measured in patients with ED and maintained at adequate levels by specific supplementation in case of deficiency. However, the low quality and heterogeneity of clinical trials evaluating the effects of vitamin D administration on erectile function and ED-associated comorbidities do not allow for a univocal conclusion, and indicate the need for further studies to analyze these aspects.

## 1. Introduction

Erectile dysfunction (ED) is the inability to achieve or maintain sufficient penile stiffness for satisfactory sexual intercourse and is a leading cause of sexual dysfunction affecting hundreds of millions of men worldwide [1]. It negatively influences interpersonal relationships and the quality of life of affected men and their partners [2]. It also causes a significant economic burden, as ED patients have decreased productivity at work even when controlling for potential confounders [3]. Epidemiologic studies regarding the prevalence of ED have demonstrated a strong positive association between this condition and age [4]. Indeed, an umbrella review of 98 meta-analyses reported a prevalence of approximately 20% in men younger than 30 years of age, 25% in men 30–39 years of age, 40% in 40–49 years of age, 60% among 50–59-year-olds, 80% in 60–69 year-olds, and 90% in men over 70 years old [5].

The pathophysiology of ED depends on an alteration of the psychosocial and organic physiologic factors that occur in a normal erection. In a normal physiologic erection, nitric oxide (NO) released from the endothelium and nerve terminals causes cavernous and arterial smooth muscle relaxation. The resulting arterial dilation increases the flow to the cavernous sinusoids, which compress the emissary veins within the rigid tunica albuginea [6]. Therefore, a reduction in NO production or sensitivity may cause ED. For example, in vasculogenic ED, endothelial dysfunction due to vascular disease reduces endothelial NO levels and sensitivity to this signaling molecule [7].

ED can be broadly classified as organic or psychogenic, but most cases have both components [8]. Vasculogenic ED is the most common cause of organic ED and is particularly prevalent in older men and those with risk factors for heart and vascular disease such as hypertension, dyslipidemia, diabetes mellitus (DM), atherosclerosis, obesity, cigarette smoking, and other cardiovascular disorders [8].

Vitamin D is a steroid hormone and vitamin that plays a critical role in regulating calcium-phosphorous homeostasis and affects the proper functioning of multiple organ systems [9]. The Endocrine Society defines vitamin D deficiency (VDD) as a 25-hydroxyvitamin D [25(OH)D] level below 20 ng/mL and insufficiency as a level between 21 and 29 ng/mL [10].

Hypovitaminosis D has an estimated prevalence of 20–100% among elderly men and women in the United States, Canada, and Europe [11]. Regarding ED, age and vitamin D levels have been linked, with increasing age being associated with reduced vitamin D levels and increased risk of hypovitaminosis D [12]. This is probably due to the decreased capacity of aging skin to produce cholecalciferol, a precursor to 25(OH)D and fully active 1α,25-dihydroxy vitamin D3 [1,25(OH)_2_D3] [12].

Other risk factors for VDD are related to ultraviolet-mediated production of vitamin D or dietary intake of vitamin D. In healthy adult men, these risk factors include decreased outdoor activities, excessive sunscreen, and reduced consumption of vitamin D-fortified milk [9]. Dark-skinned people are also at a higher risk due to the increased absorption of ultraviolet B by melanin [13].

The age dependence of hypovitaminosis D and ED seems to suggest a possible relationship between the two conditions. Consistent with this theory, it was observed that in patients older than 60 years with moderate to severe ED and lower urinary tract symptoms (LUTS), 25(OH)D levels and VDD were the independent risk factors for ED in the multivariate regression analysis, whereas these associations were not observed in analyses performed in other subgroups according to age and LUTS severity [14].

Many possible mechanisms linking VDD and ED have been proposed. In detail, the effect of VDD on endothelial function, the role of VD in the upregulation of NO synthase (NOS), the increase in the degree of inflammation, and the possible endocrinological dysregulation due to VDD are among the main mechanisms involved [15].

Several studies have shown a link between decreased vitamin D levels and ED. One study found that a significant proportion of ED patients have VDD, particularly among patients with arteriogenic ED, as assessed by penile Doppler ultrasound [16]. Wu and colleagues echoed these results by demonstrating that serum levels of 25(OH)D were related to ED severity and vasodilation function [17]. Another study also established the association between VDD and the prevalence of ED independent of atherosclerotic cardiovascular disease (CVD) risk factors. However, this study assessed ED with a self-reported response to a single question. However, the subjective assessment of ED by answering a single question is a major limitation of this study [18].

Meta-analytic studies on this topic seem to confirm this association. Indeed, in a meta-analysis of seven studies, it was observed that vitamin D levels were higher in patients without ED than in those with ED. Furthermore, the presence of VDD correlated with lower scores of the International Index of Erectile Function (IIEF)-5 scores [19]. Similarly, another meta-analysis of eight studies showed a relationship between VDD and ED severity as measured by the IIEF-5 questionnaire [20]. It should be considered, however, that both meta-analyses include studies with high heterogeneity due to the different populations involved and also to the different methods of vitamin D measurement that could influence the results [19,20].

All these studies suggest VDD could be an independent risk factor for organic ED, although more intervention studies are needed to better establish a true link between these two conditions.

In this review, we summarize the mechanisms and evidence linking vitamin D with ED. Furthermore, to provide an accurate representation of this theme, we have outlined the meta-analytic evidence on the different sub-topics analyzed.

## 2. Vitamin D and Endothelial Function

The association between ED and CVD is very close. Indeed, more than 70% of patients with CVD (including coronary artery disease, cerebrovascular disease, and peripheral artery disease) have ED [21]. Furthermore, according to the artery size theory (small size of the cavernous arteries compared to other vascular districts), ED may only be the “tip of the iceberg” of chronic vascular disease and could represent its first manifestation [22]. Consequently, the onset of ED would precede the onset of major cardiovascular events by up to several years, proving to be a low-cost biomarker for the diagnosis and prevention of CVD [21]. The close association between ED and CVD is also demonstrated by the common risk factors between the two conditions (obesity, DM, hypercholesterolemia, and hypertension). Furthermore, both conditions share endothelial dysfunction as the main pathogenic mechanism [21].

Several mechanisms contribute to the genesis of endothelial dysfunction, such as the reduced activity of endothelial NOS (eNOS) enzyme and, therefore, NO production, increased levels of reactive oxygen species (ROS) and pro-inflammatory cytokines (IL 1, TNFα), increased vascular permeability, increased vascular stiffness, and inability to regenerate from endothelial progenitor cells (EPC) [23]. Testosterone levels also play a role in this context. In fact, this hormone seems to regulate NO production, the stiffness of the vessels, and the production of inflammatory cytokines. Furthermore, hormone deficiency is associated with greater severity of the atherosclerotic process [23]. In agreement, low testosterone levels have been observed to play a predictive role in cardiovascular mortality and morbidity [24]. Vitamin D would appear to have a regulatory role in endothelial function by intervening in many of the mechanisms mentioned above, including the production of testosterone (discussed in detail in the section “Vitamin D and erectile dysfunction: The role of testicular function”). Furthermore, meta-analytical studies have observed an inverse correlation between 25(OH)D levels and cardiovascular risk, reinforcing the hypothesis of the role of this vitamin in proper vascular function [25,26].

The association between vitamin D and endothelial function is also highlighted by the correlation between vitamin D levels and some markers of endothelial dysfunction. For example, a correlation has been observed between vitamin D levels and mean platelet volume (MPV) [27]. This correlation between platelets and vitamin D is not surprising since the vitamin D receptor (VDR) is expressed at the mitochondrial level in human platelets, suggesting that this hormone may play a role in platelet differentiation and function [28]. MPV, in turn, correlates with increased cardiovascular risk and ED, as larger platelets show greater reactivity and thrombogenicity [29]. In agreement, a study of 78 patients with stable coronary artery disease, divided into three subgroups according to vitamin D levels, observed higher MPV values in patients with more severe VDD, suggesting that the regulation of thrombosis, hemostatic processes, and platelet function is another mechanism by which vitamin D affects cardiovascular function [30]. Similarly, another study of 90 patients with ED reported that the severity of ED (evaluated by the IIEF-EF questionnaire containing six items) correlates with lower vitamin D levels and that, in turn, patients with severe ED have higher MPV values. The correlation analysis showed that vitamin D levels correlated negatively with MPV values, reinforcing the hypothesis of a relationship between these two parameters [31].

Another marker of endothelial dysfunction closely associated with the presence of ED is EPC. These cells are involved in the repair of endothelial damage and have been considered progenitor cells that are found in high numbers in patients with arterial ED [32]. In this context, vitamin D has been shown to play a protective role in EPCs, probably through the inhibition of inflammation. Furthermore, it would seem to favor the correct process of migration and adhesion of these cells, thus facilitating the restoration of the integrity of the endothelial barrier [33]. Moreover, another in vitro study showed that incubation with vitamin D increases the angiogenic and, therefore, the reparative capacity of EPCs, probably by increasing vascular endothelial growth factor and pro-metalloproteinase-2, two key factors for correct angiogenesis [34].

Another marker closely associated with the previous one is the endothelial microparticles (EMPs). These are released into the circulation by endothelial cells, and their quantity is significantly higher when the endothelium is dysfunctional. Once in circulation, EMPs have pro-inflammatory and procoagulant effects, which facilitate the progression of vascular damage [35]. Among the mechanisms believed to be related to endothelial dysfunction is the increased production of ROS [36]. In vitro incubation with calcitriol was observed to prevent EMP formation from endothelial cells exposed to increased oxidative stress, probably through an increase in eNOS expression and inhibition of Rho-associated coiled-coil protein kinase 1, a key regulator of actin–myosin contraction, which appears to be abnormally expressed in patients with endothelial dysfunction and CVD [36]. Furthermore, vitamin D also seems to exert a direct antioxidant effect due to the reduction of H_2_O_2_ through the modulation of monoamine oxidases and pro-oxidant enzymes of the nitrogen oxides family [37].

Another marker of endothelial dysfunction is endothelial cell-specific molecule-1 (ENDOCAN), which is associated with systemic inflammation, DM, atherosclerosis, hypertension, and acute coronary syndrome [38]. Levels of this marker are associated with worse peak systolic velocity on dynamic penile echo color Doppler in patients with ED, and this correlates with the severity of the condition [38]. Again, vitamin D may play a role, as in healthy men, low vitamin D levels have been associated with elevated ENDOCAN levels, suggesting that VDD may contribute to endothelial damage [39]. Furthermore, vitamin D supplementation was associated with a reduction in ENDOCAN levels in patients undergoing kidney transplants, suggesting its role as therapy in reducing endothelial dysfunction [40].

Other evidence in favor of the role of vitamin D on endothelial function comes from studies that evaluated its relationship with other markers of endothelial health, such as carotid artery intima–media thickness (CIMT) [41], ankle–brachial index [42], and flow-mediated dilation (FMD) [43]. The latter is closely linked to endothelial function and is significantly reduced in patients affected by vascular diseases [43]. Consequently, a study of 100 men (50 with acute myocardial infarction (MI) and 50 age-matched healthy controls) observed that FMD and vitamin D levels were markedly reduced in patients with MI compared with controls. Furthermore, the results of this study showed the presence of a positive correlation between vitamin D levels and FMD [43]. Another double-blind randomized clinical study of 44 hypertensive and type 2 diabetic patients divided into two groups, with 23 patients treated with 2000 IU of cholecalciferol daily for 12 weeks and 21 with placebo for 12 weeks, showed that FMD increased significantly in the vitamin D-treated group. Furthermore, these patients also showed a significant reduction in oxidized LDL levels. Therefore, this study would confirm the role of vitamin D in the reduction of endothelial dysfunction [44]. Similarly, an association was observed between vitamin D levels, CIMT, and ED, suggesting that the assessment of vitamin D levels may be useful in the diagnosis of ED and that its supplementation may play a role in therapeutic management [41].

In addition to acting on the endothelium by promoting NO production, reducing platelet aggregation, modulating the coagulation cascade by reducing tissue factor levels, and upregulating thrombomodulin and antithrombin, vitamin D also counteracts inflammation [45]. This, in turn, is one of the main mechanisms of endothelial dysfunction and the atherosclerotic process [23]. In detail, vitamin D can regulate the function of both innate and adaptive immune responses. For the innate response, it regulates antigen-presenting dendritic cells, promoting the formation of immature dendritic cells that express fewer major histocompatibility complex (MHC) class II molecules and costimulatory molecules and are therefore less able to present the antigen. This is associated with decreased IL12 production and increased IL10 expression resulting in modulation of the immune response [46]. Regarding the adaptive response, vitamin D seems to favor the transition from a Th1 inflammatory response to a Th2 anti-inflammatory response as it reduces the production of Th1 CD4-positive lymphocytes, favors the Th2 response through the induction of IL4, IL5, and IL10, and suppresses the Th17 response favoring the production of regulatory T cells [46]. As further evidence of its anti-inflammatory effects, vitamin D serum levels have been observed to correlate negatively with the neutrophil/lymphocyte ratio (NLR) [47]. This parameter is a well-known marker of inflammation that has been seen to be increased in CVD but also significantly increased in patients with ED [48], which negatively correlates with the severity of ED [49]. An RCT performed on a large cohort of adolescent girls showed that high-dose vitamin D supplementation (50,000 units weekly for 9 weeks) is able to reduce NLR values and, thus, systemic inflammation [50]. However, to our knowledge, no studies have also evaluated this in patients with ED.

Despite the ample evidence in favor of the role of vitamin D on endothelial function, interventional studies evaluating the effects of supplementation on markers of endothelial function have yielded conflicting results and most often have failed to demonstrate any relationship [51]. Furthermore, meta-analytic studies that have collected evidence from interventional studies have also yielded conflicting results [52,53,54,55,56,57] and, in most cases, have not found an association between vitamin D supplementation and endothelial function [53,54,55,56] or found a correlation only in selected patients [57] (Table 1).

## 3. Vitamin D and Erectile Dysfunction: Relationship with Diabetes Mellitus

ED is a common ailment in diabetic patients. A recent meta-analysis of 145 studies involving 88,577 men showed that the overall prevalence of ED among diabetic male patients is 52.5%, particularly 37.5% for type 1 DM, 66.3% for type 2, and 57.7% for both types of DM. Additionally, diabetic men have a 3.62 higher odds ratio of having ED than healthy controls [58]. These data underscore the importance of ED screening for male diabetic patients.

Several mechanisms relate DM to ED. Indeed, hyperglycemia is responsible for angiopathic and neuropathic damage, which are key mechanisms in the pathogenesis of ED. In detail, hyperglycemia causes an increase in the production of ROS, which interferes with endothelial function and favors the inflammatory and pro-thrombotic processes [59].

Evidence from the literature suggests that vitamin D is able to improve glycemic and metabolic profiles. Therefore, this could be another mechanism through which vitamin D intervenes in the pathogenesis of ED. Indeed, it is assumed that vitamin D is involved in insulin secretion from pancreatic ß-cells and the tissue response to insulin [60] since VDRs are expressed in pancreatic ß-cells, adipocytes, and, probably, also in myocytes [60]. Considering these findings, it has been suggested that vitamin D plays a role in insulin sensitivity and secretion, and its supplementation improves glucose tolerance and prevents or delays the progression to DM [61]. Moreover, VDD could participate in the pathogenesis of DM, considering the role of this vitamin in regulating sub-inflammation, one of the main factors involved in the physiopathology of the metabolic syndrome [62]. Additionally, vitamin D status may contribute to the development of diabetic complications, such as nephropathy, retinopathy, neuropathy, and atherosclerosis [63].

Vitamin D can modulate insulin resistance through both direct and indirect mechanisms. As regards the direct mechanism, as already mentioned, vitamin D has a role in the secretion of insulin by pancreatic ß-cells. Indeed, calcitriol treatment leads to increased cytoplasmic calcium levels in insulin-secreting MIN6 cells, and in animal studies, promotes the biosynthetic capacity of ß-cells and the conversion of proinsulin to insulin [61]. As regards the indirect mechanism, vitamin D, by acting on the regulation of intra- and extracellular calcium levels, can modulate the dephosphorylation of glucose transporter 4 and, consequently, the insulin-stimulated glucose transport [61]. Furthermore, a vitamin D-responsive element has been found in the promoter of the human insulin receptor gene, and the expression of this gene increases after calcitriol administration [64], suggesting another mechanism by which vitamin D can affect insulin sensitivity. Finally, vitamin D may act directly or indirectly on enzymes involved in mitochondrial metabolism, as the oxidation/utilization ratio of glucose at high glucose levels was reduced in VDD ß-cell islets [65]. In general, several in vitro animal studies have shown glucose-induced impaired insulin secretion in VDD animals [65]. Notably, mice with a lack of VDR function have a 60% decreased insulin secretion after glucose loading [66].

In addition to a direct role in blood glucose regulation, other mechanisms seem to confirm a correlation between vitamin D and ED in diabetic patients. In fact, it has been observed that the administration of calcitriol reduces platelet aggregation in patients with DM, suggesting the role of this vitamin in the treatment of DM-associated vascular complications [67]. Similarly, the presence of VDD has been associated with EPC depletion in diabetic patients, which, as aforementioned, are known to play a key role in the regeneration of endothelial damage [68].

Several studies have evaluated the relationship between vitamin D levels and DM and/or prediabetes. In particular, the association between VDD and DM types 1 and 2 has been studied with conflicting results. For example, Afzal and colleagues conducted a prospective cohort study, finding an association between lower vitamin D concentration and a higher cumulative prevalence of type 2 DM. The same authors conducted a meta-analysis on 16 studies, whose results support their findings, with an odds ratio of 1.5 for type 2 DM for the lower versus the higher quartile of 25(OH)D [69]. Another randomized Mendelian analysis aiming to establish whether the association between low vitamin D levels is causal or not evaluated the polymorphisms (SNPs) related to four genes (DHCR7, CYP2R1, DBP, and CYP24A1) involved in the biosynthesis of vitamin D, hepatic 25-hydroxylation, transport, and catabolism, respectively. It was found that SNPs were not associated with the risk of developing type 2 DM, and there was no significant association between 25(OH)D levels and the risk of type 2 DM [70]. Conversely, other meta-analytic studies have confirmed an inverse relationship between circulating 25(OH)D levels and the prevalence of prediabetes [71] or DM [72,73].

Similarly, the usefulness of vitamin D supplementation in glucose metabolism is still controversial. A 2017 meta-analysis investigated this aspect and found no differences in fasting blood glucose (FBG) between diabetic vitamin D-treated and untreated patients. Instead, they found a modest reduction in HbA1c after vitamin D treatment, although the heterogeneity of the studies may invalidate these findings [74]. A recent double-blind, randomized, placebo-controlled clinical trial involving 162 patients with prediabetes and hypovitaminosis D evaluated the effects of high-dose vitamin D on fasting plasma glucose, 2 h oral glucose tolerance test, and the assessment of the Homeostatic Model Assessment of Insulin Resistance (HOMA-IR). The results demonstrated a significantly lower HOMA-IR in the vitamin D-treated group compared to the placebo group, suggesting that vitamin D supplementation improves insulin sensitivity. Furthermore, this study showed a reduced rate of progression to DM in the vitamin D group [75]. Consistent with this finding, meta-analytic studies have found that vitamin D supplementation in prediabetic patients reduces the risk of progression to DM and increases the reversion rate of prediabetes to normoglycemia [76], probably by reducing FBG, fasting insulin, and HbA1c [77]. Even Li and colleagues, in their systematic review and meta-analysis, found that vitamin D supplementation reduced insulin resistance in patients with type 2 DM as this treatment significantly improved HOMA-IR [78]. They also noted that these effects were most evident with a high dose of vitamin D over a short period in normal-weight, Middle Eastern, and VDD patients or those with optimal glycemic control at baseline [78]. The authors also suggested potential influencing factors on the effects of supplementation, such as baseline vitamin D status, HbA1c, body mass index, and ethnicity, highlighting the need for more studies [78]. The same study showed no significant effect on fasting insulin [78]. Similar results were found by two other meta-analyses demonstrating that vitamin D supplementation significantly reduces not only the HOMA index, but also FBG and HbA1cin patients with type 2 DM [79,80]. Furthermore, in one of these meta-analyses, it was found that even a minimum dose of 4000 IU/day is able to induce these effects [80]. In contrast, another meta-analysis does not suggest improvements in FBG [71,81], HOMA-IR [71,82], or HbA1c [71,83]. Table 2 summarizes the meta-analyses evaluating the correlation between vitamin D levels and the prevalence of DM and the effects of vitamin D supplementation on the glycemic profile.

To date, only one meta-analysis of four studies has evaluated the role of vitamin D in patients with DM and ED, noting that 25(OH)D levels in these patients are significantly lower than in diabetic patients without ED, thus suggesting a role of VDD in the pathogenesis of ED in diabetic patients [84].

## 4. Vitamin D and Erectile Dysfunction: Relationship with Blood Hypertension

The association between hypertension and ED is well known. In fact, 41% of ED patients were observed to have hypertension versus 19% in age-matched men without ED, confirming that ED patients are more likely to have hypertension [85]. Similarly, a meta-analysis of eighteen cross-sectional studies involving a total of 41,943 participants and 10,151 ED patients found that the risk of ED is higher in patients with hypertension than in controls even after adjusting for confounders, suggesting thus that hypertension is a risk factor for ED [86]. Several mechanisms are involved in this association. In fact, a hypertensive state favors the release of pro-contractile factors, compromising the normal balance between vasoconstrictor and vasodilator substances, and also favors the production of ROS, causing endothelial dysfunction [87]. Furthermore, a role has also been demonstrated for the innate immune system, mainly mediated by Toll-like receptor 4, which in turn is involved in the genesis of oxidative stress and a low-grade inflammatory state [87]. Finally, antihypertensive drugs can also cause ED [88].

Another mechanism by which vitamin D might influence the pathogenesis of ED is through the regulation of blood pressure. Furthermore, this action on pressure is not only exerted through the regulation of endothelial function. Indeed, an independent association between vitamin D levels and elements of the renin–angiotensin–aldosterone system (RAAS) has been demonstrated, indicating a regulatory and suppressive role of the vitamin in this system [89]. In detail, 1–25 hydroxy-vitamin-D would activate its receptor, which in turn would bind the cyclic adenosine monophosphate (cAMP)-response element-binding protein (CREB), causing a block of the promoter activity of the gene for renin, which would therefore be less expressed [90]. In agreement, Carrara and colleagues showed in a population of 33 patients with hypertension and hypovitaminosis D that vitamin D supplementation at a dose a 50,000 IU/week for eight weeks was associated with a reduction in plasma renin activity (PRA), renin levels, and angiotensin II levels [91]. The authors also found that vitamin D supplementation improves FMD, although this improvement is not due to changes in the RAAS system. This appears to confirm that vitamin D is able to regulate blood pressure not only by regulating RAAS but also through direct stimulation of eNOS in endothelial cells or by reducing inflammatory cytokines and nuclear factor kappa-light-chain-enhancer of activated B cells (NF-κB) [91].

Another mechanism by which vitamin D affects blood pressure levels is the regulation of insulin resistance. Indeed, there would seem to be a close association between hypertension and insulin resistance. Both conditions often occur together in the context of metabolic abnormalities. However, the association should not only be considered casual, but a direct cause-and-effect relationship has also been proposed [92]. In fact, insulin resistance seems to promote hypertension through the increase in sodium reabsorption in the renal tubules, the activation of the sympathetic nervous system, and the alteration of vascular resistance by increasing the concentration of calcium in smooth muscle cells [92]. Since it has been observed that vitamin D supplementation may reduce insulin resistance [93], treatment of this condition may be another mechanism by which vitamin D may help reduce blood pressure. According to this theory, a meta-analysis of 26 RCTs on diabetic patients showed that vitamin D supplementation was able to reduce systolic blood pressure values, especially in patients with vitamin D values below 50 nmol/L [94].

Among other mechanisms, a role for parathyroid hormone (PTH) has also been proposed. In fact, in patients with VDD, there is an increase in the levels of PTH, which seem to act by promoting atherosclerotic processes and, therefore, vessel rigidity [95]. Furthermore, low calcium levels associated with hypovitaminosis D could also contribute to the hypertensive phenomenon as adequate calcium levels reduce RAAS activity, regulate sodium–potassium balance, and inhibit vascular smooth muscle cell contraction [95]. Finally, Vitamin D exerts vasodilation and anti-atherosclerotic activity by stimulating prostacyclin secretion from smooth muscle cells and regulating the growth, differentiation, and migration of vascular smooth muscle cells [95].

The relationship between vitamin D levels and the risk of hypertension has been demonstrated by numerous meta-analytic studies, which all seem to agree on this aspect [96,97,98,99,100,101]. In detail, a recent meta-analysis of 11 cohort studies and 43,320 participants showed an inverse association between hypertension and vitamin D levels, with a 7% reduction in the risk of hypertension for every 25 nmol/L of increase in serum vitamin D levels [96]. Similarly, another meta-analysis including prospective and cross-sectional studies for a total of 70 studies showed that in prospective studies, patients with high serum vitamin D levels had a 16% decreased risk of hypertension compared to those with low levels. Furthermore, a dose–response analysis suggested that each 25 nmol/L increase in serum vitamin D concentrations resulted in a 5% reduction in the risk of hypertension. Meta-analysis of cross-sectional studies also showed a reduction in the odds of developing hypertension and pre-hypertension in patients with the highest versus the lowest serum vitamin D levels. The dose–response analysis of this study showed that each 25 nmol/L increase in serum vitamin D levels resulted in a 6% reduction in the odds of hypertension in all populations and a 3% in studies with representative populations [97]. Another meta-analysis, including a very large cohort of 283,537 patients, found an inverse relationship between 25(OH)D levels and the risk of hypertension, also demonstrating a 12% decrease in the risk of future hypertension for each 10 ng/mL increment in circulating 25(OH)D levels [98]. However, while the association between vitamin D levels and hypertension appears to be widely accepted, meta-analytic studies compiling data from RCTs evaluating the effects of vitamin D supplementation on blood pressure values compared to placebo have often yielded conflicting results. In detail, most studies have failed to find a correlation between vitamin D supplementation and blood pressure levels in the general population [96,99,100,101,102,103,104,105,106,107,108]. However, two meta-analyses observed an effect of supplementation only on diastolic blood pressure (DBP) [109,110] and only one on systolic blood pressure (SBP) [111]. Nevertheless, if vitamin D supplementation in the general population appears to be ineffective, when specific subpopulations are considered, the results appear to be different. Indeed, in the meta-analysis by He and colleagues, which included 17 RCTs, when considering only patients aged >50 years, there is a reduction in SBP values in patients treated with vitamin D [106]. Similarly, Golzarand and colleagues showed a reduction in both SBP and DBP in patients aged >50 years supplemented with cholecalciferol 800 IU daily for less than 6 months [105]. Furthermore, when only hypertensive and VDD patients are considered, vitamin D supplementation is able to reduce both SBP and DBP [106]. Accordingly, another meta-analysis on elderly subjects demonstrated that when only patients with VDD are considered, a reduction in SBP values was demonstrated in supplemented patients [104]. Finally, even excluding obese and overweight patients [105] or patients with previous cardiometabolic diseases [107], vitamin D supplementation seems to be effective in reducing blood pressure. All of these studies would seem to suggest that vitamin D supplementation should be carefully evaluated based on the patient’s phenotype. Table 3 summarizes meta-analyses evaluating the relationship between vitamin D levels and the risk of hypertension and the effects of vitamin D supplementation on blood pressure.

## 5. Vitamin D and Erectile Dysfunction: Relationship with Hypercholesterolemia

Hyperlipidemia is a well-known important risk factor for CVD and, for this reason, for the onset of ED [112]. Indeed, a close correlation has been observed both between low HDL cholesterol levels and ED [113,114] and between increased total cholesterol levels and ED [102]. Further evidence for the beneficial effects of statin treatment in hypercholesterolemic patients comes from meta-analytic studies. Indeed, combined treatment with statins and sildenafil was reported to improve erectile function more than combining sildenafil with a placebo [115]. Furthermore, treatment with these drugs could also be effective in the treatment of patients with ED non-responders to phosphodiesterase type 5 inhibitor administration [116]. A recent meta-analysis seems to confirm these aspects, showing that atorvastatin is able to improve erectile function in patients both alone or in combination with other treatments for ED [117].

Some evidence seems to show that vitamin D may also play a role in lipid dysmetabolism, which could therefore be another link between vitamin D and ED. Among the proposed mechanisms, VDD would seem to be particularly capable of increasing cholesterol biosynthesis. This effect would be determined by the reduced transcriptional activity of the VDR in the presence of VDD. This reduced transcriptional activity would inhibit the expression of insulin-induced gene-2 (Insig-2), which normally plays a role in the negative regulation of sterol regulatory element-binding protein 2 (SREBP-2). The latter stimulates the expression of 3-hydroxy-3-methylglutaryl-coenzyme A reductase [118]. Furthermore, a direct effect of vitamin D on SREBP-2 expression was also observed [119]. Finally, the reduction in triglycerides in differentiated adipocytes, the increase in ß-oxidation of fatty acids, and the reduction in the synthesis of new fatty acids have been proposed as possible mechanisms of action of vitamin D on the lipid profile [120]. Among the other proposed mechanisms, vitamin D increasing intestinal calcium absorption could decrease the hepatic secretion of microsomal triglyceride transfer protein that is involved in triglyceride synthesis and secretion [121]. Moreover, adequate levels of vitamin D maintain PTH levels in the normal range, while, if elevated, they can increase lipogenesis and, consequently, serum triglyceride levels [122]. Finally, insulin resistance increases the production of LDL and triglycerides and reduces the synthesis of HDL [123]. As mentioned above, vitamin D might decrease insulin resistance counteracting this mechanism [93].

Consistent with a correlation between vitamin D levels and dyslipidemia, a recent meta-analysis including 57 cross-sectional studies and 2 cohort studies observed that higher vitamin D levels were associated with a 19% decrease in the odds of hypertriglyceridemia. In addition, dose–response analyses demonstrated a 7% decrease in the likelihood of hypertriglyceridemia for each 10 ng/mL increase in serum 25(OH)D levels. Similarly, higher vitamin D levels were associated with an 18% reduction in the odds of low HDL cholesterol levels and an 18% reduction in the odds of dyslipidemia. In this case, dose–response analysis showed decreases in the odds of 3% and 4%, respectively, with each 10 ng/mL increase in the serum 25(OH)D levels [124].

Again, meta-analytical studies evaluating the effects of vitamin D supplementation on the lipid profile have produced conflicting results, probably due to the extreme heterogeneity among the populations included in the various studies. Notably, only two studies have found no effects of vitamin D administration on the lipid profile [125,126]. One study analyzed a patient population with metabolic syndrome [125] and another a patient population with non-alcoholic fatty liver disease [126]. One study analyzed the effects of vitamin D supplementation on the lipid profile of diabetic patients and found a significant improvement in total cholesterol, triglycerides, and LDL levels in treated patients [127]. Two studies analyzed the effects of vitamin D supplementation in women with PCOS and found a significant reduction in total cholesterol, triglycerides, LDL, and VLDL levels in treated women, while no effect on HDL levels [128,129]. Another study analyzed patients with chronic kidney disease and found a reduction in total cholesterol and triglycerides in patients treated with vitamin D and no effect on HDL and LDL [130]. When considering the general population, only three meta-analyses found an effect of supplementation on increasing HDL-cholesterol levels [131,132,133], although one of these found the effect only in patients treated for more than 26 weeks [133]. Three other studies observed a correlation between vitamin D supplementation and reduction in triglyceride levels, while two observed a correlation between vitamin D administration and reduced total cholesterol [131,133,134]. Finally, three meta-analyses observed the effects of LDL reduction in patients treated with vitamin D [133,134,135], although, in one of these, the reduction was observed only in patients treated with a daily dose of vitamin D greater than 400 IU [133]. Table 4 summarizes evidence from meta-analyses evaluating the relationship between vitamin D levels and dyslipidemia and the effects of vitamin D supplementation on the lipid profile.

## 6. Vitamin D and Erectile Dysfunction: Relationship to End-Stage Chronic Renal Disease

There is also a close relationship between ED and end-stage chronic kidney disease (ECKD). Indeed, in these patients undergoing dialysis, the overall prevalence of ED is approximately 71% [136]. This prevalence decreases to 59% in patients going through kidney transplantation [136], suggesting that this procedure may improve erectile function in patients with ECKD [137].

Several mechanisms explain why patients with ECKD have a high prevalence of ED. First, ECKD patients exhibit hormonal abnormalities consisting of low total testosterone levels, normal SHBG levels, and thus low free testosterone levels [138]. Furthermore, increased uric acid damages both Leydig and Sertoli cells, causing hypergonadotropic hypogonadism [138]. Finally, prolactin levels are elevated in these patients due to reduced clearance of this hormone because of renal insufficiency. This leads to a reduction in GnRH secretion, which worsens the severity of hypogonadism [138].

Among the other mechanisms, ECKD is associated with endothelial dysfunction and autonomic nervous system disorders [138]. Additionally, erythropoietin also appears to play a role. Indeed, its low levels in patients with ECKD reduce its antiapoptotic effects on the cavernous system and prolactin lowering. Moreover, anemia itself can also cause ED [138]. Higher PTH levels secondary to VDD are another possible mechanism both for his action in the atherosclerotic process [95] and for his direct role in the stimulation of prolactin secretion [126]. Finally, the role of drugs should also be considered [138].

Therefore, vitamin D could play a role in the treatment of ED in these patients. As discussed in this overview, it could act directly by improving endothelial dysfunction (see Section 2) and testicular testosterone secretion (see Section 7), and indirectly by reducing PTH levels [138]. Accordingly, several meta-analyses have evaluated the effects of supplementation with vitamin D or its derivatives on the vascular function of patients with renal insufficiency [139,140,141,142] (Table 5).

In particular, both vitamin D and paricalcitol supplementation appear to improve FMD [139,140] and reduce PWV [139]. Furthermore, paricalcitol supplementation would appear to be effective in reducing the risk of cardiovascular events in these patients [141]. Only one meta-analysis failed to find an association between vitamin D supplementation and endothelial function [142].

## 7. Vitamin D and Erectile Dysfunction: The Role of Testicular Function

Hypogonadism is an important risk factor for ED. A meta-analysis of 656 subjects demonstrated that approximately one-third of ED patients are androgen deficient [143]. At the same time, testosterone replacement therapy (TRT) improves overall sexual function in patients with ED and low androgen levels [143]. TRT improves penetration, erection maintenance, and desire in patients with ED and androgen deficiency [144]. Testosterone modulates sexual function through several mechanisms. Androgen receptors expressed in different areas of the human brain are involved in the regulation of male sexual function [145]. Thus, testosterone is important in the release of stimulatory neurotransmitters such as dopamine, oxytocin, and NO that control sexual development and behaviors, and libido [146]. Testosterone can also modulate the structure and innervation of trabecular smooth muscle cells of penile vessels and the fibroelastic properties of the corpus cavernosum [147]. It acts as a vasodilator in the penis in part by stimulating the release of NO [148].

Testosterone, which has been considered the fuel of male sexual function, is produced by Leydig cells in the testis. Interestingly, human Leydig cells also express the cytochrome P450 family 2 subfamily R member 1 (CYP2R1) gene [149], which encodes 25-hydroxylase [150]. Therefore, the testis is able to convert cholecalciferol to 25(OH)D, just like the liver. Consequently, Leydig cell dysfunction could result in both lower circulating levels of testosterone and 25(OH)D. Indeed, previous studies have shown that testicular CYP2R1 gene expression is lower in patients with testiculopathy [151].

Human Leydig cells also express VDRs [149]. Therefore, a possible direct association between hypovitaminosis D and hypotestosteronemia has been hypothesized [152]. VDR knockout mice develop hypergonadotropic hypogonadism [153]. Furthermore, in human Leydig cells, in vitro addition of 1,25(OH)_2_D_3_ up-regulates gene expression of steroidogenic enzymes involved in the production of androgens and their precursors [154]. A synergistic effect of LH and 1,25(OH)_2_D_3_ to increase testosterone synthesis has been hypothesized. Although experimental evidence has shown an association between vitamin D and testosterone, the results of epidemiological studies are still controversial.

A systematic review reported conflicting results on the effect of vitamin D supplementation on testosterone in men [155]. Consequently, a systematic review and meta-analysis of 10 randomized clinical trials (RCTs) showed that vitamin D supplementation had no significant effect on total testosterone (MD = 0.20, 95% CI: −0.20, 0.60, and *p* = 0.336) and sex hormone-binding globulin levels (MD = 1.56, 95% CI: −0.85, 3.97, and *p* = 0.204) [156]. A year later, D’Andrea and colleagues published a systematic review and meta-analysis of 18 studies evaluating the difference in total testosterone levels between men with and without VDD. A slight, albeit barely significant, positive association between 25(OH)D and total testosterone levels was found (pooled SMD: −0.23, 95% CI: −0.45 to −0.01, and *p* = 0.04) [152]. However, there was very large heterogeneity between studies (I2 = 98%, *p* for heterogeneity < 0.00001), and when studies were grouped based on the health characteristics of the patients evaluated, a positive association between vitamin D and testosterone levels was present only in men with frailty states [152]. Thus, the authors hypothesized that the association between VDD and hypotestosteronemia might reflect non-biological correlates but could be related to common underlying causes and risk factors. Indeed, low circulating levels of vitamin D are a common finding in people with chronic comorbidities, especially in the elderly. At the same time, testosterone levels decrease with age in men [157] and patients with chronic diseases [158]. Therefore, further large-scale RCTs are needed to evaluate the association between vitamin D and testosterone levels in men.

Besides the secretion of sex hormones, the other fundamental function of testicular function is sperm production. VDR has been discovered in the smooth muscles of the epididymis, spermatogonia, and Sertoli cells of male rodents, suggesting a role for vitamin D in spermatogenesis [159]. In 2006, the presence of VDR was also confirmed in human spermatozoa, predominantly expressed in the sperm head [160].

Several studies have also looked at the association between vitamin D and sperm parameters. However, even with regard to this aspect, the results are not clear. A recent systematic review and meta-analysis showed that 25(OH)2D3 levels are significantly higher in fertile men than in infertile patients (WMD −0.63; 95% CI, −1.06 to −0.21, and *p* = 0.003) [161]. The authors also reported a significant association between serum 25(OH)D, sperm total motility (WMD −5.84; 95% CI, −10.29 to −1.39, and *p* = 0.01), and sperm progressive motility (WMD −5.24; 95% CI, −8.71 to −1.76, and *p* = 0.003) [161]. However, even these findings should be taken with caution due to significant heterogeneity among included studies [161]. Adamczedwska and colleagues, in a recent systematic review, emphasized the lack of robust evidence to support the use of vitamin D supplementation to improve outcomes for patients with abnormal sperm parameters or hormonal dysfunction [162]. Consequently, also based on the study by Arab and colleagues, the authors reported the beneficial effect of vitamin D on sperm quality, especially sperm motility. However, more high-quality RCTs are needed to better elucidate the association between vitamin D and testicular function.

Table 6 summarizes evidence from meta-analyses evaluating the association between vitamin D and testicular function.

## 8. Limits

When interpreting data from studies analyzing the effects of vitamin D levels and its supplementation on outcomes of interest, several limitations must be considered. Indeed, as discussed in the various sections of this review, meta-analyses often obtain conflicting results. This is probably due to the extreme heterogeneity of the included studies, which differ in the type of population analyzed, the amount of vitamin D administered, and also the duration of treatment. Furthermore, it should be considered that in most studies, the evaluation of vitamin D is performed with immunometric assays, which have been shown to be less accurate with a variability of 10 to 30 percent, which may compromise the definition of VDD status. In this regard, it is now established that an adequate method to measure serum vitamin D levels, especially in populations at high risk of deficiency, must be performed by liquid chromatography-mass spectrometry [163]. Therefore, further studies using proper vitamin D measurement methods conducted in well-selected populations are needed to investigate the correlation between vitamin D levels and outcomes of interest. Finally, in the case of studies with vitamin D supplementation, a careful evaluation of the dosages and duration of treatment is necessary. Additionally, any further meta-analyses should take these aspects into account in the interpretation of the results to minimize the impact of heterogeneity between studies.

## 9. Conclusions

**Figure 1:**
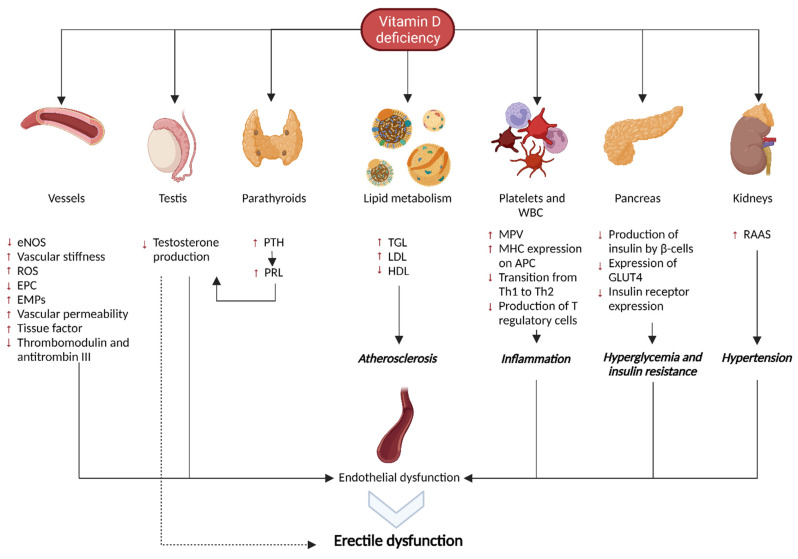
Mechanisms correlating vitamin D deficiency with erectile dysfunction. List of Abbreviations: LUT4 = Glucose Transporter Type 4; EMPc = endothelial microparticles; eNOS = endothelial nitric oxide synthase; EPC = endothelial progenitor cells; HDL = High-density lipoproteins; LDL = Low-density lipoproteins; MHC = major histocompatibility complex; MPV = mean platelet volume; PTH = parathormone; PRL = prolactin; RAAS = renin angiotensin aldosterone system; ROS = reactive oxygen species; TGL = triglycerides; and Th = T-helper.

Albeit with limitations, vitamin D would seem to be a useful therapeutic target to be evaluated in patients with ED, given its close correlation with endothelial and testicular function and with the main comorbidities associated with ED (Figure 1).

To date, only a few studies have evaluated vitamin D administration as a possible therapeutic strategy to treat patients with ED [164,165]. Indeed, a retrospective study demonstrated a greater improvement in erectile function in patients with ED treated with tadalafil and vitamin D (4000 IU/day) compared to those treated with tadalafil alone [164]. Similarly, another study observed that patients with LUTS and ED who were unresponsive to treatment with 5 mg of tadalafil after 1 month of adding 100,000 IU/week of vitamin D therapy had a marked improvement in IIEF-5 questionnaire score from the baseline [165]. However, further well-designed randomized clinical trials are needed to better evaluate the efficacy of vitamin D administration, as well as the dose to use and the duration of treatment.

## Figures and Tables

**Table 1:** Meta-analyses that evaluated the effects of vitamin D supplementation on endothelial function.

First Author and Year of Publication	N° of Studies Included and Study Design	Total Sample Size	Outcome Assessed	Main Findings
Beveridge et al., 2018 [56]	31 RCTs	2751	Effects of vitamin D supplementation on FMD, plethysmography, AI PWV, central aortic BP, acetylcholine iontophoresis, and laser Doppler perfusion imaging	Vitamin D supplementation showed no improvement in these parameters
Hussin et al., 2017 [53]	16 RCTs	1177	Effects of vitamin D supplementation on endothelial function measured by ultrasound, venous occlusion plethysmography, photoplethysmography, pulse wave velocity, pulse amplitude tonometry, and laser Doppler flowmetry	No correlation between EF and vitamin D supplementation
Pincombe et al., 2019 [54]	26 RCTs	2808	Effects of vitamin D supplementation on endothelial function assessed by FMD, PWI, and AI	Vitamin D supplementation showed no improvement in EF
Tabrizi et al., 2018 [52]	21 RCTs	1604	Effects of vitamin D supplementation on endothelial function assessed by FMD, PWI, and AI in patients with metabolic syndrome and related disorders	Vitamin D supplementation seems to improve FMD, but it does not seem to affect PWI and AI
Stojanovic et al., 2015 [57]	8 RCTs	529	Effects of vitamin D supplementation on FMD	Supplementation with vitamin D did not improve FMD, with exception of patients with high SBP
Upala et al., 2016 [55]	7 RCTs	547	Effects of vitamin D supplementation on PWV and AI	Vitamin D supplementation showed no improvement in these parameters

Abbreviations: AI = augmentation index; BP = blood pressure; EF = endothelial function; FMD = flow-mediated dilation; PWI = pulse wave index; PWV = pulse wave velocity; RCTs = randomized clinical trials; and SBP = systolic blood pressure.

**Table 2:** Meta-analyses evaluating serum Vitamin D levels’ relationship with prediabetes and diabetes mellitus prevalence and the effect of vitamin D supplementation on glycemic profile.

First Author and Year of Publication	N° of Studies Included and Study Design	Total Sample Size	Outcome Assessed	Main Findings
Afzal et al., 2012 [69]	14 observational studies	72,204	Association between levels of 25(OH)D and risk of DM2	The odds ratio for DM2 was 1.50 for the bottom vs. the top quartile of 25(OH)D
Forouhi et al., 2012 [73]	11 prospective studies	59,325	Association between levels of 25(OH)D and risk of DM2	Inverse association between circulating 25(OH)D and incident DM2
Krul-Poel, 2016 [83]	23 RCTs	1797	The effect of vitamin D supplementation on glycemic control in patients with DM2	No significant effect in change in HbA1c after VD intervention compared with placebo. A favorable effect of VD on fasting glucose in patients with poorly controlled diabetes
Lee et al., 2017 [74]	22 RCTs	2747	Effect of vitamin D supplementation on glucose metabolism	Modest reduction (−0.32%) in HbA1c in patients treated with VD compared to placebo, greater in those who reached the repletion of vitamin D deficiency (although not significant). No overall differences were found in FBG between the two groups
Li et al., 2018 [78]	20 RCTs	2703	Effect of vitamin D supplementation on glucose metabolism	No difference in FBG between intervention and control groups. Insignificant difference in the reduction of HbA1c between the two groups. No significant effect on fasting insulin. Larger reduction of HOMA-IR in the intervention group
Mirhosseini et al., 2017 [80]	24 prospective clinical trials	1528	The effect of vitamin D supplementation and improved vitamin D status on glycemia and insulin resistance in DM2	Vitamin D supplementation, at a minimum dose of 4000 UI/die, may significantly reduce serum FPG, HbA1c, and HOMA index and could also help to control glycemic response and improve insulin sensitivity in patients with type 2 diabetes
Poolsup et al., 2016 [82]	10 RCTs	1718	The effect of vitamin D supplementation on insulin resistance and glycemic control in prediabetes	No beneficial effect of vitamin D in improving insulin resistance was identified
Sahebi et al., 2019 [79]	37 (27 RCTs and 10 cross-sectional studies)	1673	The effects of vitamin D supplementation on HOMA-IR, FBG, HbA1c, QUICKI (quantitative insulin-sensitivity check index), and lipid profile in diabetic patients	Vitamin D supplementation improves FBG, HOMA-IR, and HbA1C in patients with DM
Song et al., 2013 [72]	21 prospective studies	76,220	Association between 25(OH)D blood levels and incident risk of DM2	Inverse and significant association between circulating 25(OH)D levels and risk of DM2 was found
Wu et al., 2017 [81]	24 RCTs	1874	Effect of vitamin D supplementation on glycemic control in patients with DM2	VD supplementation reduces HbA1c, but has no influence on FBG. However, in patients with 25(OH)D deficiency at baseline, its supplementation reduces FBG, and in patients with BMI < 30, HbA1c is significantly reduced after vitamin D supplementation
Yu et al., 2020 [71]	4 observational studies	3094	The association between serum levels of 25(OH)D and prediabetes	Low serum 25(OH)D levels are associated with a high risk of prediabetes
	8 RCTs	865	Differences in therapeutic effects between patients with prediabetes treated with vitamin D and those treated with placebo	No significant differences in change in HbA1c, HOMA-IR, or FBG between prediabetic patients treated with vitamin D and those treated with placebo; whereas significant differences in change were found in plasma glucose after 2 h oral glucose tolerance test
Zhang et al., 2020 [76]	8 RCTs	4896	Effect of vitamin D supplementation on the risk of DM2 in patients with prediabetes	In patients with prediabetes, vitamin D supplementation reduces the risk of progression to diabetes and increases the reversion rate of prediabetes to normoglycemia
Zhang et al., 2021 [77]	29 RCTs	3792	Effect of vitamin D supplementation on glycemic controls in patients with prediabetes	Oral supplementation of vitamin D improves FBG, HbA1c, and fasting insulin compared with controls among prediabetic patients. Long-term vitamin D supplementation could have additional effects in participants with vitamin D deficiency for 2h-PG, HOMA-IR, and HOMA-B (homeostasis model assessment of β-cell function)

Abbreviations: BMI = body mass index; t2dm = diabetes mellitus type 2; FBG = fasting blood glucose; HbA1c = glycated hemoglobin; RCTs = randomized clinical trials; and 25(OH)D = 25-hydroxy-Vitamin D.

**Table 3:** Meta-analyses evaluating the relationship between vitamin D levels and risk of hypertension and the effects of vitamin D supplementation on blood pressure.

First Author and Year of Publication	N° of Studies Included and Study Design	Total Sample Size	Outcome Evaluated	Main Findings
Burgaz et al., 2011 [101]	4 prospective and 14 cross-sectional studies	78,028	Correlation between 25(OH)D status and risk of HTN	Inverse correlation between 25(OH)D levels and HTN
Elamin et al., 2011 [102]	14 RCTs	1518	Effects of vitamin D supplementation on SBP and DBP	No effects of vitamin D supplementation in DBP and SBP
Farapti et al., 2020 [104]	12 RCTs	2468	Effects of vitamin D supplementation on SBP and DBP in elderly	No difference between vitamin D supplementation group and placebo group. However, when only VDD patients are considered, there is a significant reduction in SBP
Golzarand et al., 2016 [105]	30 RCTs	4744	Effects of vitamin D supplementation on SBP and DBP	Vitamin D supplementation has no effect on SBP and DBP in the general population. However, when obese and overweight patients are excluded, vitamin D supplementation reduces both SBP and DBP. Moreover, daily vitamin D3 therapy at a dose of >800 IU/day for <6 months in subjects ≥50 years old reduced both SBP and DBP
He et al., 2019 [106]	17 RCTs	1687	Effects of vitamin D supplementation on SBP and DBP in patients with VDD	No difference in SBP and DBP between vitamin D supplementation group and controls. However, when patients with age >50 years are considered, there is a significant reduction in SBP. In patients with both VDD and hypertension, vitamin D supplementation reduces both SBP and DBP
Jafari et al., 2018 [94]	26 RCTs	1798	Effects of vitamin D supplementation on SBP and DBP in diabetic patients	Vitamin D supplementation reduces SBP, particularly in patients with levels < 50 nmol/L
Kunutsor et al., 2013 [98]	8 observational studies	283,537	Correlation between 25(OH)D status and risk of HTN	Inverse association between baseline circulating levels of 25(OH)D and risk of HTN. Decrease in 12% of the risk of future HTN for every 10 ng/mL increment in circulating 25(OH)D levels
Kunutsor et al., 2014 [107]	16 RCTs	1879	Effects of vitamin D supplementation on SBP and DBP	No effects of vitamin D supplementation on SBP and DBP, with the exception of reduction in DBP in patients with pre-existing cardiometabolic disease
Mokhtari et al., 2022 [97]	70 cohort and cross-sectional studies	32,7701	Serum 25(OH)D levels in relation to HTN and pre-HTN in adults	Serum 25(OH)D concentrations are inversely related to the risk of HTN in adults in a dose–response manner
Morvaridzadeh et al., 2020 [110]	8 RCTs	17,644	Effects of calcium and vitamin D co-supplementation on SBP and DBP	No effects on SBP but significant reduction in DBP
Pittas et al., 2010 [100]	3 cohort studies	32,181	Correlation between 25(OH)D status and risk of HTN	Association between low 25(OH)D levels and risk of HTN
	9 RCTs	37,162	Effects of vitamin D supplementation on SBP and DBP	No effects of vitamin D supplementation on DBP and SBP
Qi et al., 2016 [103]	8 RCTs	917	The effect of vitamin D supplementation on HTN in non-CKD populations	Vitamin D is not an antihypertensive agent, although it has a moderate SBP-lowering effect
Qi et al., 2017 [99]	7 prospective studies	53,375	Correlation between 25(OH)D concentrations and incident HTN	Lower serum 25(OH)D concentrations were associated with a greater risk of incident HTN
Witham et al., 2020 [109]	8 RCT	475	Effects of vitamin D supplementation on SBP and DBP	No effects of VD on SBP, while there is slight reduction in DBP
Wu et al., 2010 [111]	4 RCTs	429	Effects of vitamin D supplementation on SBP and DBP	VD supplementation reduces SBP but not DBP
Wu et al., 2017 [108]	8 RCTs	36,806	Effects of calcium plus vitamin D co-supplementation on SBP and DBP	No effects of vitamin D on SBP and DBP
Zhang et al., 2020 [96]	11 cohort studies	43,320	Correlation between 25(OH)D levels and risk of HTN	Inverse relationship between 25(OH)D levels and risk of HTN
	27 RCTs	3810	Effect of vitamin D supplementation on BP and HTN in the general population	Supplementation with vitamin D does not lower BP in the general population

Abbreviations: BP = blood pressure; CKD = chronic kidney disease; DBP = diastolic blood pressure; HTN = hypertension; RCTs: randomized clinical trials; SBP = systolic blood pressure; VDD = vitamin D deficiency; and 25(OH)D = 25-hydroxy-Vitamin-D.

**Table 4:** Meta-analyses evaluating the relationship between vitamin D levels and dyslipidemia and the effects of vitamin D supplementation on lipid profile.

First Author and Year of Publication	N° of Studies Included and Study Design	Total Sample Size	Outcome Evaluated	Main Findings
AlAnouti et al., 2020 [125]	4 RCTs	226	Effect of vitamin D supplementation on serum lipid profiles in patients with metabolic syndrome	No effects of vitamin D supplementation on serum lipid profile
Bahadorpour et al., 2022 [124]	57 cross-sectional	210,575	Relationship between serum 25(OH)D levels and dyslipidemia	25(OH)D levels are inversely related to the odds of hypertriglyceridemia, low HDL, and dyslipidemia in a dose–response manner. However, no significant association was observed for high serum LDL or hypercholesterolemia
	2 cohort studies	8494		
Dibaba et al., 2019 [134]	41 RCTs	3434	Effect of vitamin D supplementation on serum lipid profiles	Vitamin D supplementation improved serum TC, LDL cholesterol, and TGL but not HDL cholesterol levels
Jafari et al., 2016 [127]	17 RCTs	1356	Effect of vitamin D supplementation on serum lipid profiles in DM2	Vitamin D supplementation improved serum levels of TC, TGL, and LDL in patients. Moreover, baseline serum 25(OH)D of patients, vitamin D dosage, and intervention duration influence the effect of vitamin D supplementation on lipid profile
Jin et al., 2020 [129]	8 RCTs	467	Effect of vitamin D supplementation on serum lipid profiles in PCOS patients	Significant reduction in TGL, TC, LDL, and VLDL levels after supplementation. No effect on HDL
Luo et al., 2021 [128]	11 RCTs	667	Effect of vitamin D supplementation on serum lipid profiles in PCOS patients	Significant reduction in TGL, TC, LDL, and VLDL levels after supplementation. No effect on HDL
Milajerdi et al., 2019 [130]	6 RCTs	323	Effect of vitamin D supplementation on serum lipid profiles in patients with CKD	Reduction in TGL and TC levels after supplementation. No effects on HDL and LDL
Morvaridzadeh et al., 2021 [131]	13 RCTs	2304	Effect of vitamin D and calcium co-supplementation on serum lipid profiles	Significant reduction in TGL and TC levels and increase in HDL levels after supplementation
Ostadmohammadi et al., 2019 [132]	8 RCTs	630	Effect of vitamin D supplementation on serum lipid profiles in patients with CVD	Significant increase in HDL-cholesterol in patients supplemented with vitamin D
Tabrizi et al., 2017 [126]	7 RCTs	452	Effect of vitamin D supplementation on serum lipid profiles in patients with non-alcoholic fatty liver disease	No effect of vitamin D supplementation on TGL, LDL, and TC
Wang et al., 2012 [135]	12 RCTs	1346	Effect of vitamin D supplementation on serum lipid profiles	Significant reduction in LDL levels. No effects on the other parameters
Zhang et al., 2022 [133]	7 RCTs	1109	Effect of vitamin D supplementation on serum lipid profiles in post-menopausal women	Vitamin D supplementation decreases TGL. Moreover, it increases HDL levels when the treatment is under 26 weeks and decreases LDL for doses higher than 400 UI/die

Abbreviations: CVD = cardiovascular disease; DM = diabetes mellitus; HDL = high-density lipoprotein; LDL = low-density lipoprotein; PCOS = polycystic ovary syndrome; RCT = randomized clinical trials; TC = total cholesterol; TGL = triglycerides; VLDL = very low-density lipoprotein; and 25(OH)D = 25-hydroxy-Vitamin-D.

**Table 5:** Meta-analyses evaluating the effects of vitamin D supplementation on vascular function of patients with chronic kidney disease.

First Author and Year	N° of Studies Included and Study Design	Total Sample Size	Outcome Assessed	Main Findings
Dou et al., 2019 [139]	7 RCTs	429	Effects of VD supplementation on FMD, PWV, SBP, and DBP in patients with CKD	Supplementation with Cholecalciferol and 2 mcg of paricalcitol was able to improve FMD; supplementation with cholecalciferol reduced PWV, with no effect on SBP and DBP
Hu et al., 2018 [141]	21 RCTs	1894	Effects of paricalcitol supplementation on cardiovascular risk of patients affected by CKD	Paricalcitol supplementation reduces cardiovascular events compared to placebo
Hu et al., 2020 [142]	10 RCTs	579	Effects of VD supplementation on FMD, PWI, SBP, DBP, and CRP in patients with CKD	Vitamin D supplementation showed no improvement in these parameters
Lundwal et al., 2018 [140]	4 RCTs	305	Effects of VD supplementation on FMD in patients with CKD	Short-term intervention with VD is associated with improvements in EF, as measured by FMD

Abbreviations: CKD = chronic kidney disease; CRP = C-reactive protein; DBP = diastolic blood pressure; EF = endothelial function; FMD = flow mediate dilation; PWV = pulse wave velocity; RCTs = randomized clinical trials; SBP = systolic blood pressure; and VD = vitamin D.

**Table 6:** Meta-analyses evaluating the association between vitamin D and testicular function.

First Author and Year	N° of Studies Included and Study Design	Total Sample Size	Outcome Assessed	Main Findings
Arab et al.; 2019 [161]	18 observational studies	4773	Association between vitamin D and sperm parameters Difference in serum levels of vitamin D in fertile and infertile subjects	25(OH)D was significantly associated with sperm motility but not with other sperm parameters 1,25 (OH)_2_D_3_ was significantly higher in fertile men compared to infertile patients
D’Andrea et al., 2020 [152]	18 observational studies	9892 men with vitamin D deficiency 10,675 controls	Difference in circulating TT levels between men with and without vitamin D deficiency	A slight, albeit just significant, positive association between 25(OH)D and TT levels was found. However, a very large heterogeneity between the studies was found
Marnani Hosseini et al., 2019 [156]	10 RCTs	1061	Effect of vitamin D supplementation on TT and SHBG levels	Vitamin D supplementation had no significant effect on TT and SHBG

Abbreviations: RCTs = randomized clinical trials; TT = total testosterone; SHBG = sex hormone binding globulin; 25(OH)D = 25-hydroxy-Vitamin-D; and 1,25(OH)_2_D3 = 1α,25-dihydroxyvitamin D3.

## Data Availability

Not applicable.
